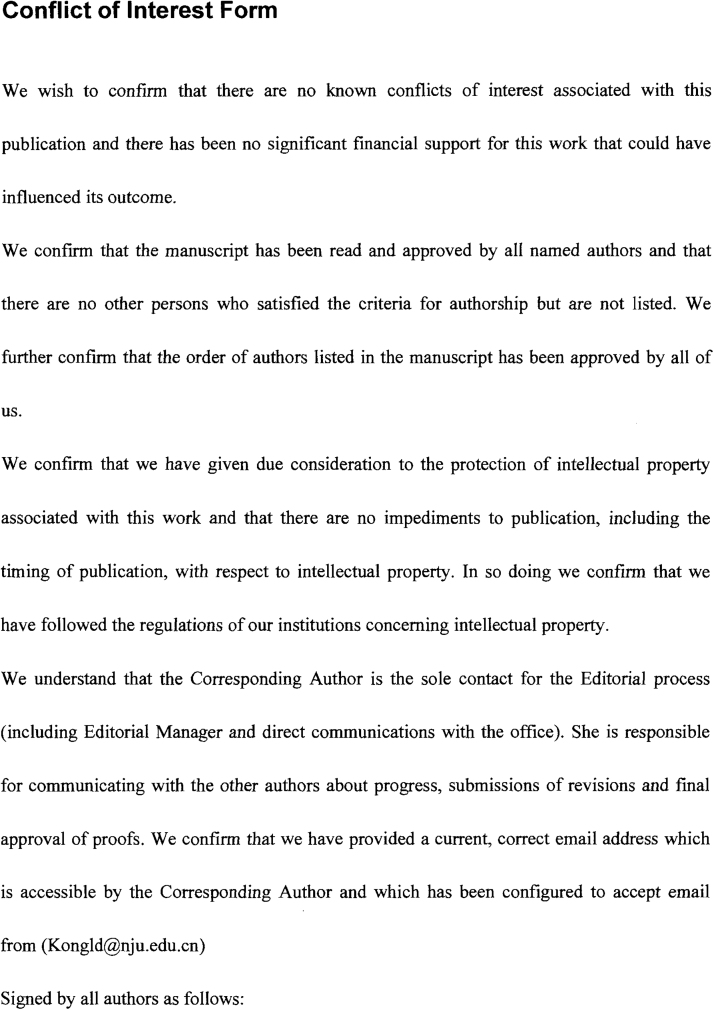# Dataset on assessment of magnesium isoglycyrrhizinate injection for dairy diet and body weight in fructose-induced metabolic syndrome of rats

**DOI:** 10.1016/j.dib.2018.03.016

**Published:** 2018-03-08

**Authors:** Xiao-Juan Zhao, Yan-Zi Yang, Yan-Jing Zheng, Shan-Chun Wang, Hong-Mei Gu, Ying Pan, Shui-Juan Wang, Hong-Jiang Xu, Ling-Dong Kong

**Affiliations:** a State Key Laboratory of Pharmaceutical Biotechnology, School of Life Sciences, Nanjing University, Nanjing 210023, PR China; b Drug Screening and Evaluation Department of R&D Institute, Chia Tai Tianqing Pharmaceutical Group Co., LTD, Nanjing 210023, PR China

## Abstract

The data presented herein are related to the research article entitled “Magnesium isoglycyrrhizinate blocks fructose-induced hepatic NF-κB/NLRP3 inflammasome activation and lipid metabolism disorder” (Zhao et al., 2017) [1]. This article describes the effects of magnesium isoglycyrrhizinate on 24-h food or water intake in fructose-fed rats at 15-week. In addition, this article expands the effect of magnesium isoglycyrrhizinate on the animal body weight change during 1–17 week. The field dataset is made publicly available to enable critical or extended analyzes.

**Specifications Table**

**Table:** 

Subject area	*Biology*
More specific subject area	*Pharmacological Study on Metabolic Diseases*
Type of data	*Table*
How data was acquired	*Raw data are recorded during the animal experiment.*
Data format	*Raw*
Experimental factors	*Male Sprague-Dawley rats consumed drinking water or 10% fructose solution in drinking water (weight/volume) for 6 weeks. Fructose-fed rats were treated intraperitoneally with saline injection, or 10, 20 and 40* *mg/kg magnesium isoglycyrrhizinate injection, or intragastrically 4* *mg/kg pioglitazone for the last 11 weeks (n = 8–10/group), respectively.*
Experimental features	*For 24-h food and water intake:*
	*Rats were housed in metabolic cages with free access to standard chow and water. The 24-h food and water intake was recorded, respectively.*
	*For body weight:*
	*Animal body weight was detected weekly.*
Data source location	*State Key Laboratory of Pharmaceutical Biotechnology, Nanjing University, Nanjing, Nanjing 210023, PR China*
Data accessibility	*The data are available with this article*

**Value of the data**

• The data reported here demonstrate the effects of magnesium isoglycyrrhizinate injection on 24-h food and water intake, and animal body weight in high fructose-fed rats.
• The data provide some reference for researchers who investigate the effects of magnesium isoglycyrrhizinate injection on rat food and water intake, and body weight.
• This data allows other researchers to extend the statistical analyses.

## 1 Data

This article describes 24-h food and water intake in Control-Vehicle, Fructose-Vehicle, 10, 20 and 40 mg/kg magnesium isoglycyrrhizinate- and 4 mg/kg pioglitazone-treated rats with fructose exposure after 15 weeks. Additionally, animal body weight change was described during 1–17 weeks. Table 1 shows 24-h food and water intake. Table 2 shows animal body weight.

**Table 1:** Assessment of magnesium isoglycyrrhizinate injection on 24-h food and water intake in fructose-fed rats.

Group	24-h food intake (g)	24-h water intake (ml)
Control + vehicle	10.7	21
	0.9	15
	2.6	15
	2.7	25
	1.6	14
	9.1	4
	7.9	13
	8.0	21
Fructose + vehicle	3.2	15
	5.8	17
	4.8	19
	0	14
	3.0	4
	1.5	13
	10.0	21
	4.7	14
Fructose + 10 mg/kg magnesium isoglycyrrhizinate	5.1	35
	4.5	15
	6.1	21
	1.2	12
	0	7
	7.2	16
	2.4	8
	0.4	9
	3.2	17
	5.9	16
Fructose + 20 mg/kg magnesium isoglycyrrhizinate	0.4	16
	0.3	22
	1.1	12
	0.8	20
	0	10
	0	7
	0.7	11
	0	13
	7.5	12
	3.8	30
Fructose + 40 mg/kg magnesium isoglycyrrhizinate	0.3	13
	0.4	8
	1.3	9
	0.5	40
	2.7	15
	0	10
	2.5	7
	0.6	8
	1.6	7
	0.8	6
Fructose + 4 mg/kg pioglitazone	2.0	6
	6.0	11
	6.0	8
	0	7
	2.9	17
	0.7	25
	2.8	6
	5.6	10

**Table 2:** Assessment of magnesium isoglycyrrhizinate injection on body weight (g) in fructose-fed rats.

Time (week)	Control vehicle	Fructose + Vehicle	Fructose + Magnesium isoglycyrrhizinate Fructsoe + Pioglitazone (mg/kg)			
			10	20	40	4
1	204.1	201.4	211.2	192.4	209.7	206.0
	188.6	201.4	200.9	202.4	206.6	204.0
	203.3	214.7	193.1	191.4	209.0	205.1
	191.3	194.7	201.5	193.8	207.7	201.3
	189.1	212.4	196.4	204.4	198.6	198.0
	188.7	203.7	201.3	202.3	203.5	199.3
	190.3	193.4	199.2	200.2	203.5	204.8
	202.3	193.5	189.3	208.9	200.3	203.5
			209.7	203.3	199.7	
			195.6	200.7	196.5	
2	241.0	271.5	261.9	259.4	248.4	236.5
	255.5	251.6	251.0	274.7	239.4	251.8
	240.2	272.2	232.7	257.2	243.7	237.4
	247.4	241.4	257.5	262.3	242.7	253.1
	254.5	258.4	273.4	259.9	239.0	271.6
	247.7	252.7	274.7	250.1	252.3	252.3
	240.4	248.9	274.5	231.3	247.9	251.2
	232.1	248.5	273.2	264.3	247.4	262.7
			274.5	272.3	273.5	
			250.9	257.5	273.2	
3	286.0	299.1	299.3	293.7	275.3	312.3
	275.0	316.3	288.6	325.0	255.5	289.0
	282.1	320.7	287.5	277.4	271.6	300.6
	278.3	283.7	291.3	298.4	259.5	279.1
	272.5	302.2	316.5	315.4	283.6	292.6
	260.9	277.0	333.9	289.1	374.5	310.7
	281.2	293.7	324.2	260.2	323.9	314.5
	283.9	293.7	321.6	315.9	305.7	332.4
			331.4	303.0	319.0	
			295.4	295.9	308.7	
4	371.9	314.4	328.8	319.4	304.4	340.0
	303.7	310.8	319.7	372.9	276.6	314.5
	319.3	333.6	318.7	330.0	303.9	323.1
	313.1	356.8	322.3	329.5	280.5	303.3
	288.4	344.4	359.4	300.5	309.4	328.3
	319.0	337.7	369.0	346.7	346.5	336.5
	304.3	318.4	366.5	347.3	323.1	368.1
	312.5	313.7	346.2	334.3	315.7	352.5
			360.5	340.0	344.2	
			330.9	337.4	332.4	
5	356.1	367.3	354.9	350.2	344.3	372.7
	350.2	368.6	340.8	396.2	305.0	343.5
	342.4	397.9	333.2	311.3	332.4	350.2
	347.7	344.4	350.4	362.5	311.3	310.4
	350.4	368.3	391.2	363.5	3234.2	336.5
	320.0	348.2	401.7	345.6	386.7	344.0
	337.7	339.4	391.8	321.8	346.8	392.1
	360.5	343.6	371.4	377.3	330.7	366.6
			392.4	377.4	377.4	
			362.5	371.5	355.3	
6	372.9	400.7	415.1	415.8	407.7	351.9
	367.6	367.7	368.7	436.0	355.3	406.5
	360.6	419.4	342.5	406.6	381.3	379.4
	381.71	396.9	392.6	429.2	370.0	377.5
	360.4	364.4	357.8	386.8	361.3	369.1
	377.7	372.1	380.3	381.9	396.2	428.3
	382.4	401.6	372.5	384.8	375.1	401.4
	338.0	361.4	340.4	406.6	367.1	366.3
			422.4	396.5	387.7	
			375.2	392.8	386.6	
7	393.7	448.2	412.1	367.4	456.3	399.8
	384.0	403.4	399.2	360.2	408.7	363.8
	379.7	420.6	388.1	384.7	412.3	395.6
	398.1	381.4	403.0	370.6	468.1	360.6
	384.1	398.7	439.8	409.2	380.7	395.4
	395.4	402.8	463.9	368.6	414.7	464.1
	409.2	460.3	447.6	456.7	417.6	404.8
	356.1	434.3	434.2	438.0	410.3	402.6
			457.6	389.0	387.2	
			353.1	434.0	441.0	
8	391.2	445.9	447.0	465.3	389.5	457.4
	401.9	396.3	412.9	419.3	389.5	457.4
	391.1	449.2	428.3	385.3	379.3	416.8
	406.6	468.7	424.8	426.1	356.9	455.9
	406.6	431.8	427.0	449.0	389.1	411.7
	405.2	393.6	443.3	428.8	476.0	405.8
	427.4	422.7	483.9	410.8	418.3	484.9
	366.1	397.8	438.7	421.1	408.2	354.3
			472.6	417.6	459.3	
			390.2	424.5	409.4	
9	434.7	424.5	469.4	486.9	466.1	403.9
	444.3	492.0	455.9	435.4	437.7	521.9
	415.0	470.8	473.4	405.3	438.1	439.9
	418.4	475.4	440.4	449.1	416.0	445.1
	433.4	413.7	459.2	473.1	442.2	416.2
	391.3	420.2	431.7	445.0	437.1	463.2
	455.0	446.3	418.3	406.2	500.0	499.4
	431.5	466.4	436.7	425.5	413.9	
			497.6	450.8	478.1	
			415.4	468.7	432.1	
10	431.0	486.2	448.3	487.2	468.8	449.9
	456.9	437.2	485.6	467.7	444.9	542.8
	433.3	486.3	518.7	419.4	532.8	455.7
	452.8	504.4	497.7	460.6	436.9	520.6
	449.5	470.7	487.0	502.0	399.1	463.9
	442.2	429.1	434.1	467.5	445.7	427.3
	470.5	468.0	475.1	423.5	427.4	477.1
	400.0	443.5	459.3	455.7	381.3	
			509.7	474.1	491.7	
			433.2	463.7	437.7	
11	432.2	436.4	454.4	487.7	473.2	470.8
	465.9	492.1	494.3	462.5	454.4	447.1
	421.8	513.5	523.8	419.0	556.9	543.0
	443.6	514.0	509.8	463.9	451.9	474.8
	453.9	477.3	496.3	521.4	443.1	536.5
	446.4	427.9	464.8	481.8	474.2	424.4
	455.7	474.8	439.8	451.4	457.7	451.2
	392.6	457.0	467.8	476.3	411.3	
			499.2	465.6	424.7	
			433.9	495.1	454.7	
12	455.8	507.1	463.7	526.8	491.2	495.1
	508.5	455.9	492.3	488.6	477.9	473.6
	472.8	523.6	527.6	431.1	579.0	564.3
	481.5	527.9	493.6	482.0	464.5	570.2
	476.2	508.1	495.4	513.5	458.9	502.4
	446.0	464.3	455.4	489.4	478.5	469.0
	495.0	489.8	471.8	461.1	473.7	536.1
	422.3	463.6	475.2	492.7	454.4	
			560.9	499.9	516.9	
			443.7	461.3	469.8	
13	451.7	493.5	463.9	434.7	494.6	501.1
	510.3	472.3	501.1	484.0	471.0	474.6
	463.1	527.6	542.4	516.8	593.6	566.3
	481.3	553.3	515.2	515.0	468.4	584.4
	455.3	499.6	495.1	531.7	473.4	509.6
	480.2	446.4	463.8	502.2	504.2	483.2
	507.6	502.3	474.3	471.0	492.0	518.3
	425.1	465.3	473.8	516.9	472.1	
			557.9	452.5	488.7	
			510.9	476.3	463.2	
14	462.5	535.7	461.4	458.3	502.4	506.6
	529.6	476.9	523.9	508.6	477.5	482.4
	487.3	553.3	544.9	525.6	625.4	604.9
	478.8	576.5	543.8	535.6	492.3	596.4
	469.1	527.0	523.8	546.1	486.3	521.2
	500.5	472.2	482.4	518.8	531.8	491.9
	529.7	501.3	498.9	472.5	496.4	537.3
	437.8	465.6	508.0	532.4	504.8	
			578.5	501.3	549.8	
			519.1	457.1	473.7	
15	465.7	550.0	468.8	475.2	519.2	508.7
	530.4	493.8	533.9	526.4	481.5	500.0
	482.1	558.6	555.9	541.2	632.4	625.9
	490.4	578.5	541.6	549.0	502.4	584.5
	477.6	504.8	534.2	542.1	475.9	513.0
	471.2	460.0	480.2	504.8	518.6	482.4
	541.0	525.5	492.7	475.8	480.6	525.6
	448.0	478.4	508.9	523.6	479.2	
			572.7	452.9	560.0	
			507.4	505.6	487.3	
16	513.9	531.1	475.6	486.8	535.5	512.5
	453.6	481.3	565.2	535.3	483.3	497.3
	467.4	556.7	558.3	550.6	636.1	608.7
	494.0	588.4	538.2	548.1	522.8	567.9
	483.5	512.1	561.2	543.5	509.1	510.1
	465.0	454.5	485.9	531.6	560.1	525.8
	445.2	530.8	503.5	487.1	500.0	540.2
	529.1	472.4	531.0	524.0	502.5	
			588.0	449.6	552.4	
			530.4	507.6	488.7	
17	460.2	523.6	478.3	491.1	501.2	521.4
	532.4	489.4	545.4	528.0	467.8	485.6
	484.0	568.6	554.0	540.5	606.1	610.3
	509.3	597.3	545.8	536.6	513.8	572.9
	503.3	532.7	548.3	560.1	495.7	526.7
	475.8	473.0	483.2	523.9	551.2	528.8
	537.6	556.3	511.2	496.8	499.3	527.4
	450.7	481.3	519.7	553.5	504.1	
			581.2	452.1	541.5	
			524.6	478.5	478.3	

## 2 Experimental design, materials and methods

### 2.1 Materials

Fructose was provided from Shandong Xiwang Sager Industry Co., Ltd. (Binzhou, China). Pioglitazone table was got from Jiangsu DeYuan Pharmaceutical Co., Ltd. (Lianyungang, China, Lot number: 150,792). Magnesium isoglycyrrhizinate injection was obtained from Chia Tai Tianqing pharmaceutical group Co., Ltd. (Nanjing, China, Lot number: 150,819,104 and 150,829,204).

### 2.2 Experimental design and methods

Male Sprague-Dawley rats (180–220 g) were purchased from Experimental Animal Center of Zhejiang province (Hangzhou, China) (Production license: SCXK 2014-0001). These animals were maintained at a temperature of 22 ± 2 °C and relative humidity (55 ± 5%) with a 12-h light/dark cycle. Rats were fed a standard chow and water ad libitum for the experiment and one week for acclimatization before the experiment. Each rat was given drinking water or 100 ml drinking water containing 10% fructose (weight/volume) for 6 weeks. Then these fructose-fed rats were further divided into five subgroups, receiving saline injection (by intraperitoneal injection), 10, 20 and 40 mg/kg magnesium isoglycyrrhizinate injection (by intraperitoneal injection) or 4 mg/kg pioglitazone (by intragastric administration) for additional 11 weeks (n = 8–10/group), respectively. At week 15, rats were removed to metabolic cages (one rat per metabolic cage) with free access to standard chow and water. 24-h food and water intake was recorded, respectively. Animal body weight was detected weekly.

## Figures and Tables

**Fig. S1:**
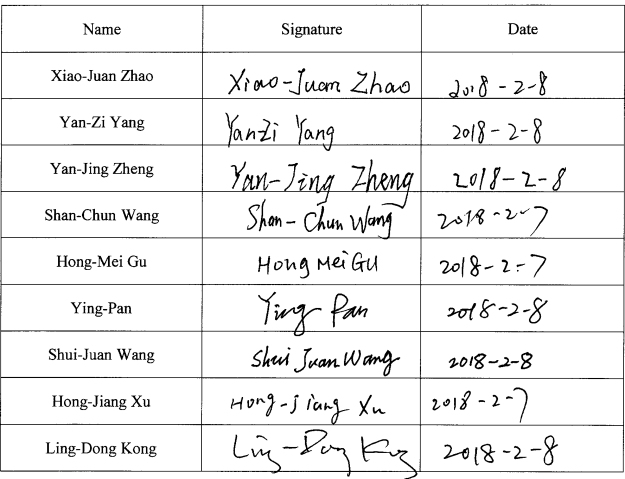


**Fig. S2:**